# Preliminary Report of the Effectiveness of Tetracycline Sclerotherapy in Treatment of Ganglion

**DOI:** 10.1155/2012/624209

**Published:** 2012-03-26

**Authors:** J. A. Ashindoitiang

**Affiliations:** Department of Surgery, Lagos University Teaching Hospital (LUTH), Idi Araba, PMB Lagos, 12003, Nigeria

## Abstract

Ganglion, a benign cyst, most common soft tissue tumor of the hand, usually occurs in the hand, wrist, and foot. It is difficult to treat as reoccurrence is common after surgery and also following other treatment procedures. In this study, sclerotherapy technique in 20 patients treated using a solution of tetracycline after aspiration is described. Most of the patients in this study were between the ages of 20 and 35 years. 16 patients had ganglion on the dorsum of the wrist, 2 patients had bilateral ganglion, and 2 patients had ganglion on the dorsum of the foot. Under aseptic conditions, the ganglions were aspirated using size 21 G needles, and then 1 mL equivalent to 100 mg/mL solution of tetracycline was injected. In the postoperative followup ranging between 18 months to 5 years, 2 reoccurrences were noticed that required the same technique. This procedure is simple, safe, effective, and cheap when compared to surgery and other nonsurgical procedure of treating ganglion.

## 1. Introduction

Ganglion accounts for two-thirds of all hand tumours [1]. The commonest site of occurrence is the dorsum of the wrist, the volar aspect of the wrist, and in the fingers in relation to the flexor tendon sheath. 

Ganglion also occurs on the dorsum of the foot. 

Surgical treatment is required when they cause symptoms. The common indications for surgery are pain or the size of the ganglion for cosmetic reasons. Recurrence after surgical excision is extremely common [2]. 

An alternative to surgical excision is aspiration with or without injection of local steroids and various other techniques [3, 4].

This is a preliminary report of the result of aspiration followed by instillation of tetracycline as a sclerosant. This procedure is simple, cheap, and has several advantages which outweigh the benefit of surgery. The advantages are the following.

1. No need for anesthesia, local or general.
2. No scar and hence better cosmetic result.
3. It is an office procedure without any complication such as hand edema and peripheral nerve injuring, and above all it is cost effective when compared to cost of surgical therapy.

## 2. Patients and Methods

This prospective study commenced in September 2002 and is still ongoing. This study had approval of the hospital ethical committee in accordance with the Helsinki Declaration of 2010. The patients in this study have been followed up for a minimum of 18 months and some up to 5 yrs with no sign of recurrence. The major complaint was cosmetic as well as pain.

Each patient has his or her ganglion aspirated with 5 mL syringe and 21 G needle. The gelatinous mucous material was aspirated. The content was completely empty, and this was evident by the slight bleeding at the end of procedure. While maintaining the needle in the cavity of the ganglion, 1 mL of tetracycline equivalent to 100 mg/mL (i.e., a capsule of 500 mg with 5 mL of water for injection, properly constituted) was injected. The needle is withdrawn, and a crepe bandage was applied. The same amount of sclerosant 1 mL was used regardless of the size of the ganglion. Again it was one brand of tetracycline from the same manufacturer (emzor pharmaticeuticals) that was used, hence there may be little or no variation in the contents. I am not aware of the previous use of tetracycline sclerotherapy for ganlion and it was its sclerosant properties that were exploited for probable benefit in this study.

The entire patients were given nonsteroidal anti-inflammatory drugs as analgesic because of the initial burring effect. 

Patients were seen at the SOPD 72 hours after therapy when the bandage was removed. 

There were then followup for a minimum of 18 months to see if there is any recurrence.

## 3. Results

A total of 20 patients have so far been treated in the last 5 years. There were 14 females (70%) and 6 males (30%), and age range was between 20–70 years. 

Most patients were between ages of 20 to 35 years, and most of the ganglion occured in the dorsum of the right wrist joint 16/20 (80%). 2 patient 2/20 (10%) had bilateral ganglion. 2 patients (10%) had ganglion on the dorsum of the foot (ankle joint) anteriorly. 

18 (90%) patients had sclerotherapy once with good result. 2 patients had a repeat therapy before the ganglion was successfully treated. 

Both patients were those who had recurrence after surgical excision.

## 4. Discussion

Despite the benign nature of a ganglion, it can be problematic [5]. Ganglia are the most common soft tissue tumors of the hand and wrist, comprising about 50 to 70% of all tumors of this anatomic area [6, 7], These tumors are usually filled with mucin and are attached to the adjacent joint capsule or tendon sheath.

They are usually single and can affect any joint of the hand, wrist, and foot, but the dorsum of the wrist is the most common location as seen in this study. 

Ganglion is prevalent during the second and third decades of life, and this study has similar findings: 19 out of 20 patients were in the late twenties and early thirties only one patient was 70 yrs. 

Women are more affected than men, this is reflected in this study, 14 out of 20 (70%) were females. It is said to occur with equal frequency in the dominant and assertive hand [6, 7]. However, in the study the right hand accounts for over 80% of the patients.

The indications for treatment are cosmetic, pains, and weakness of the hand. Nonsurgical treatment such as digital pressure, steroid, lidocaine injections, simple aspiration, heat, radiation, or sclerotherapy have been reported to be either not effective, with recurrence rates as high as 60%, or have limited success [8–11].

In this study, sclerotherapy with tetracycline has success rate that surpassed the general rates quoted for this form of treatment. So far 90% of the patients had sclerotherapy once with a good outcome. 10% had reoccurrence which occur within 3 months but was successfully treated at the second attempt.

The failure in these two patents is either as a result of previous surgery which altered the cyst wall or as result of the technical fault as this technique has been refined.

Several agents have been used in sclerotherapy. These include phenol, 4 hyaluronidase, 2 and OK 432 [5] with modest success. The use of tetracycline is an accepted treatment for pleural effusion, and it obliterates the pleural space and prevents reoccurrence. The effectiveness of tetracycline as a sclerosant depends on exciting inflammatory reaction between two endothelial surfaces. It was this property that was exploited in the treatment of ganglion. Tetracycline sclerotherapy appears to be most effective than other sclerosant previously used. However, one should be cautious in women of child bearing age to rule out pregnancy because of effect of tetracycline on the fetus although systemic effect from this procedure appears to be minimal.

Moreover, it is easily available, and the quality can be easily standardized and reconstituted even in the rural areas. 

This treatment is easily available and can be carried out by any doctor in contrast to surgery where good outcome depends on surgical experience, technique, and expertise. 

It is recommended that, when sclerotherapy is used, a maximum of 3 attempts should be done, before failure is assumed [12, 13]. In this study only 2 patients require the 2nd sclerotherapy, hence it is not yet failure of therapy. 

This study is preliminary findings; it will be expanded with more cases and a longer followup to establish the effectiveness of tetracycline sclerotherapy.

In conclusion, tetracycline sclerotherapy is judged to be very effective, easily available, requires little or minimal expertise, and is cheap and cost effective when compared to surgery. The success rate also appears to surpass surgery.
